# Relationships of Cigarette Smoking and Alcohol Consumption to Metabolic Syndrome in Japanese Men

**DOI:** 10.2188/jea.JE20100043

**Published:** 2010-09-05

**Authors:** Yumiko Nakashita, Masakazu Nakamura, Akihiko Kitamura, Masahiko Kiyama, Yoshinori Ishikawa, Hiroshi Mikami

**Affiliations:** 1 Osaka Medical Center for Health Science and Promotion, Osaka, Japan; 2 Department of Health Promotion Science, Division of Health Sciences, Osaka University Graduate School of Medicine, Suita, Osaka, Japan

**Keywords:** metabolic syndrome, cigarette smoking, alcohol consumption, cross-sectional study, Japan

## Abstract

**Background:**

Cigarette smoking is an important, aggravating factor in metabolic syndrome (MetS). In addition, some studies have reported that MetS is related to alcohol consumption irrespective of the amount consumed. However, the relationship of the combination of the 2 habits to MetS has not been fully described.

**Methods:**

In this cross-sectional survey, a questionnaire was used to collect information on cigarette smoking and alcohol consumption from 3904 Japanese men aged 20 years or older. MetS was defined according to Japanese criteria. Logistic regression analysis was used to analyze relationships of cigarette smoking and alcohol consumption with MetS, after adjustment for potential confounding factors.

**Results:**

Among the subjects, 581 (14.9%) had MetS. Daily cigarette and alcohol consumption were significantly associated with the prevalence of MetS (*P* < 0.0001, *P* = 0.030 for trend). The multivariate-adjusted odds ratio for the prevalence of MetS was 1.89 (95% confidence interval: 1.34–2.65) for subjects who smoked ≥30 cigarettes/day, as compared with nonsmokers; 1.54 (1.06–2.23) for those who consumed ≥69 grams of ethanol/day, as compared with nondrinkers; and 3.63 (1.91–6.90) for those who smoked ≥30 cigarettes/day and consumed ≥69 grams of ethanol/day, as compared with those who neither smoked nor drank. The interaction of smoking ≥30 cigarettes/day with drinking ≥69 grams/day was 2.03 (1.02–4.01, *P* = 0.043).

**Conclusions:**

Cigarette smoking and alcohol drinking had independent relations to the prevalence of MetS. In addition, the prevalence of MetS was higher among Japanese men who smoked and drank heavily.

## INTRODUCTION

Metabolic syndrome (MetS) is a combination of several lifestyle-related clinical features,^1^ including visceral obesity, dyslipidemia, hypertension, and glucose intolerance.^2^ Cigarette smoking should not be overlooked, because it is an important, independent risk factor of coronary heart disease and an aggravating factor for MetS.^3^ Indeed, several studies have observed a close relationship between cigarette smoking and MetS.^4^^–^^7^ The overall prevalence of MetS increases for approximately 1 year after cessation of smoking; however, a longer period of cessation is associated with a lower prevalence of MetS.^8^ Five years or more after quitting cigarette smoking, the prevalence of MetS was similar to that of nonsmokers.^9^

Findings on the relation between alcohol consumption and MetS are inconsistent. Some reports have shown that the prevalence of MetS is associated with alcohol consumption, irrespective of the amount consumed^10^^,^^11^; however, several studies have reported beneficial effects of alcohol consumption on MetS.^12^^,^^13^ To the best of our knowledge, no studies have examined the association between cessation of alcohol consumption and MetS. There are reports that cigarette smokers drink alcohol more often^14^^,^^15^ and in greater quantity^16^^,^^17^; however, only a few studies have examined the effect of the combination of cigarette smoking and alcohol consumption on MetS.^18^^,^^19^

The aim of this study was therefore to explore the relationship of the components of MetS with cigarette smoking and alcohol consumption, and with the cessation of smoking and/or drinking. We also aimed to verify the hypothesis that the combination of cigarette smoking and alcohol consumption increases the prevalence of MetS.

## METHODS

### Study subjects

We conducted a cross-sectional study of 4365 men who underwent an annual health checkup that included measurement of additional metabolic markers necessary to assess MetS. The checkups took place during the period from January through December 2008 at Osaka Medical Center for Health Science and Promotion in Japan. Most subjects were office workers. The occupational groups of subjects were managerial and supervisory (25.8%), office clerks (21.6%), sales (21.0%), technical specialists (17.6%), manufacturing (8.3%), and others (5.7%). We excluded subjects who had incomplete answers on the questionnaire or errors on check-up items (*n* = 311), were younger than 20 years (*n* = 68), or were currently being treated for cancer (*n* = 45) or mental illness (*n* = 37). Information from 3904 men was analyzed. The study protocol was approved by the Ethics Committee of Osaka Medical Center for Health Science and Promotion in Japan.

### Health questionnaire

Eating habits^20^: The questionnaire asked 19 questions about eating habits, such as “Did you often skip breakfast during the last month?” The answer choices were yes and no. The eating habit questionnaire was composed of 4 categories: overeating (4 items), fat consumption (4 items), salt consumption (6 items), and nutritional balance (5 items). One point was given for each undesirable habit.

Physical activity^21^: Subjects were asked if they were physically active more than once a week. If the answer was “yes,” we asked about the type of physical activity and its frequency. A respondent was regarded as a regular exerciser if he exercised more than 4 metabolic equivalent (MET)-hours per week.

Cigarette smoking: Subjects were asked about smoking habits (current/ex-smoker or nonsmoker). Ex-smokers were defined as those who had previously smoked cigarettes and had quit at some point before they answered the health questionnaire, which was distributed at least 3 weeks before the health check-up. Current and ex-smokers were asked about the average number of cigarette smoked per day and the duration of smoking in years. Current smokers were categorized into 4 groups by the number of cigarettes smoked per day: 1–9, 10–19, 20–29, and ≥30 cigarettes per day. Ex-smokers were categorized into 3 groups by years since cessation: <1, 1–4, and ≥5 years.

Alcohol consumption^22^: Subjects were asked about their drinking habits (current/ex-drinker or non-drinker). Ex-drinkers were defined as those who had previously drunk alcohol and had quit at some point before they answered the health questionnaire, which was distributed at least 3 weeks before the health check-up. Current and ex-drinkers were asked about the weekly frequency of alcohol consumption and the usual amount consumed daily. Current drinkers were categorized into 4 groups by the usual amount of ethanol consumed daily: 0.1–22.9, 23–45.9, 46–69, and ≥69 grams of ethanol per day. The categorization was based on *go*, a unit of measurement that equals 180 ml and is the traditional unit for Japanese *sake*, which contains 13% alcohol (equivalent to approximately 23 grams of ethanol). Ex-drinkers were categorized into 3 groups by the number of years since quitting drinking: <1, 1–4, and ≥5 years.

### Laboratory tests^23^^,^^24^

Anthropometric measurements: Height was measured with subjects’ shoes removed; weight was measured with subjects in light clothing. We calculated body mass index (BMI) as weight divided by the square of height in meters. Clinical laboratory staff measured waist circumference at the umbilical level in unclothed, standing subjects after normal expiration.^25^

Blood assays: Blood samples were drawn from the antecubital vein after overnight fasting.

Blood pressure measurements: Blood pressure was measured after subjects had rested for 5 minutes or longer in the sitting position. When systolic or diastolic blood pressure was higher than 130 or 85 mm Hg, respectively, at first measurement, blood pressure was measured again, and the second value was recorded.

### Criteria for the diagnosis of metabolic syndrome

MetS was defined using the Japanese criteria for men,^1^ ie, waist circumference ≥85 cm and at least 2 of the following components: serum triglyceride ≥150 mg/dl and/or high-density lipoprotein (HDL) cholesterol <40 mg/dl, or treatment for either elevated triglyceride or low HDL cholesterol; blood pressure ≥130/85 mm Hg or use of antihypertensive medication; fasting plasma glucose ≥110 mg/dl or use of antidiabetic medication.

### Statistical analysis

We estimated the magnitude of the association between MetS and smoking and drinking status using logistic regression analysis. Odds ratios and 95% confidence intervals (CIs) were calculated by multivariate adjustment. The multivariate adjustment included age (continuous), score for eating habits for each of the 4 groups (continuous), regular exercise (yes or no; dichotomous), and cigarette smoking or alcohol consumption status. Data were analyzed using the SPSS/PC statistical package (15.0J for Windows). A *P* value <0.05 was regarded as significant.

## RESULTS

The mean age ± standard deviation (SD) of the enrolled subjects was 46.6 ± 11.5 years, and the mean BMI was 23.6 ± 3.2 kg/m^2^. The overall prevalence of MetS was 14.9%; among current smokers and drinkers the prevalences were 34.7% and 73.2%, respectively (Table 1). Table 2 shows the numbers of subjects with MetS and its components, and the crude prevalence rates for each category of cigarette smoking and alcohol consumption status.

**Table 1.** Characteristics of study subjects

Subjects	*n* = 3904
Age (years)	46.6 ± 11.5
Height (cm)	170.2 ± 6.0
Weight (kg)	68.3 ± 10.4
Body mass index (kg/m^2^)	23.6 ± 3.2
Eating habits	
Overeating score	1.6 ± 1.0
Consumption of fats score	1.5 ± 1.1
Consumption of salt score	1.4 ± 1.3
Balance of nutrition score	2.9 ± 1.3
Regular exercise	1259 (32.2)
Cigarette smoking status	
Nonsmoker	1290 (33.0)
Ex-smoker	1260 (32.3)
Current smoker	1354 (34.7)
Alcohol consumption status	
Nondrinker	795 (20.4)
Ex-drinker	249 (6.4)
Current drinker	2860 (73.2)
Metabolic syndrome	581 (14.9)
High waist circumference	1794 (46.0)
High triglycerides	1076 (27.6)
Low HDL cholesterol	518 (13.3)
Medication for dyslipidemia	202 (5.2)
Hypertension	1452 (37.2)
Medication for hypertension	480 (12.3)
High fasting plasma glucose	146 (3.7)
Medication for hyperglycemia	142 (3.6)

HDL cholesterol: high-density lipoprotein cholesterol.

Age, height, weight, BMI, and eating habits are expressed as the mean plus standard deviation; other data are expressed as *n* (%).

**Table 2.** Number of subjects with metabolic syndrome and its components according to cigarette smoking and alcohol consumption status

	*n*	Metabolic syndrome	High waist circumference	High triglycerides	Low HDL cholesterol	Hypertension	High fasting plasma glucose
Cigarette smoking status							
Nonsmoker	1290	151 (11.7%)	505 (39.1%)	260 (20.2%)	124 (9.6%)	429 (33.3%)	41 (3.2%)
Ex-smoker	1260	208 (16.5%)	650 (51.6%)	379 (30.1%)	185 (14.7%)	597 (47.4%)	36 (2.9%)
Current smoker	1354	222 (16.4%)	639 (47.2%)	437 (32.3%)	209 (15.4%)	426 (31.5%)	69 (5.1%)
Daily cigarette consumption							
1–9 cigarettes/day	79	8 (10.1%)	29 (36.7%)	19 (24.1%)	13 (16.5%)	20 (25.3%)	2 (2.5%)
10–19	327	34 (10.4%)	131 (40.1%)	91 (27.8%)	30 (9.2%)	84 (25.7%)	15 (4.6%)
20–29	621	95 (15.3%)	291 (46.9%)	185 (29.8%)	95 (15.3%)	198 (31.9%)	32 (5.2%)
≥30	327	85 (26.0%)	188 (57.5%)	142 (43.4%)	71 (21.7%)	124 (37.9%)	20 (6.1%)
Years since cessation of smoking							
<1 year	57	10 (17.5%)	28 (49.1%)	21 (36.8%)	7 (12.3%)	21 (36.8%)	2 (3.5%)
1–4 years	322	59 (18.3%)	178 (55.3%)	112 (34.8%)	47 (14.6%)	139 (43.2%)	11 (3.4%)
≥5 years	881	139 (15.8%)	444 (50.4%)	246 (27.9%)	131 (14.9%)	437 (49.6%)	23 (2.6%)

Alcohol consumption status							
Nondrinker	795	103 (13.0%)	333 (41.9%)	194 (24.4%)	115 (14.5%)	225 (28.3%)	26 (3.3%)
Ex-drinker	249	43 (17.3%)	127 (51.0%)	75 (30.1%)	60 (24.1%)	77 (30.9%)	12 (4.8%)
Current drinker	2860	435 (15.2%)	1334 (46.6%)	807 (28.2%)	343 (12.0%)	1150 (40.2%)	108 (3.8%)
Daily alcohol consumption							
0.1–22.9 grams/day	1246	161 (12.9%)	520 (41.7%)	297 (23.8%)	168 (13.5%)	387 (31.1%)	37 (3.0%)
23–45.9	885	138 (15.6%)	428 (48.4%)	245 (27.7%)	96 (10.8%)	389 (44.0%)	38 (4.3%)
46–69	450	77 (17.1%)	231 (51.3%)	148 (32.9%)	53 (11.8%)	219 (48.7%)	18 (4.0%)
≥69	279	59 (21.1%)	155 (55.6%)	117 (41.9%)	26 (9.3%)	155 (55.6%)	15 (5.4%)
Years since cessation of alcohol consumption							
<1 year	17	2 (11.8%)	6 (35.3%)	3 (17.6%)	5 (29.4%)	3 (17.6%)	2 (11.8%)
1–4 years	91	11 (12.1%)	41 (45.1%)	21 (23.1%)	17 (18.7%)	22 (24.2%)	2 (2.2%)
≥5 years	141	30 (21.3%)	80 (56.7%)	51 (36.2%)	38 (27.0%)	52 (36.9%)	8 (5.7%)

HDL cholesterol: high-density lipoprotein cholesterol.

Table 3 shows that daily cigarette consumption was significantly associated with the prevalence of MetS (*P* < 0.0001 for trend). The multivariate-adjusted odds ratio for the prevalence of MetS in those who smoked ≥30 cigarettes per day was 1.89 (95% CI, 1.34 to 2.65), with nonsmokers as reference. Current daily cigarette consumption was significantly associated with the prevalences of high waist circumference, high triglycerides, and low HDL cholesterol; however, the odds ratio of the prevalence of hypertension was lower in current smokers than in nonsmokers.

**Table 3.** Adjusted odds ratios of the components of metabolic syndrome according to cigarette smoking status

	*n*	Metabolic syndrome	High waist circumference	High triglycerides	Low HDL cholesterol	Hypertension	High fasting plasma glucose
Cigarette smoking status							
Nonsmoker	1290	1.00	1.00	1.00	1.00	1.00	1.00
Ex-smoker	1260	1.01 (0.79–1.28)	1.05 (0.89–1.25)	1.23 (1.02–1.49)	1.34 (1.04–1.73)	1.03 (0.86–1.23)	0.63 (0.39–1.02)
Current smoker	1354	1.11 (0.87–1.41)	1.21 (1.02–1.43)	1.48 (1.22–1.79)	1.65 (1.28–2.13)	0.59 (0.49–0.71)	1.32 (0.87–2.01)

Daily cigarette consumption							
Nonsmoker	1290	1.00	1.00	1.00	1.00	1.00	1.00
1–9 cigarettes/day	79	0.78 (0.36–1.69)	0.86 (0.52–1.41)	1.18 (0.68–2.05)	2.12 (1.12–4.03)	0.53 (0.30–0.94)	0.75 (0.17–3.20)
10–19	327	0.76 (0.51–1.14)	0.92 (0.71–1.20)	1.40 (1.05–1.87)	0.93 (0.64–1.51)	0.51 (0.38–0.69)	1.30 (0.70–2.41)
20–29	621	1.06 (0.79–1.43)	1.06 (0.86–1.31)	1.38 (1.09–1.75)	1.76 (1.29–2.39)	0.60 (0.47–0.75)	1.32 (0.80–2.18)
≥30	327	1.89 (1.34–2.65)	1.44 (1.09–1.89)	2.27 (1.70–3.03)	2.86 (1.99–4.11)	0.66 (0.49–0.89)	1.44 (0.78–2.65)
*P*-value for trend^a^		<0.0001	<0.0001	0.001	<0.0001	0.197	0.509

Years since cessation of smoking							
Nonsmoker	1290	1.00	1.00	1.00	1.00	1.00	1.00
<1 year	57	1.29 (0.62–2.68)	1.22 (0.70–2.13)	2.04 (1.15–3.63)	1.26 (0.55–2.89)	0.95 (0.52–1.73)	0.96 (0.22–4.15)
1–4 years	322	1.34 (0.95–1.89)	1.57 (1.21–2.04)	1.81 (1.37–2.39)	1.54 (1.06–2.24)	1.17 (0.90–1.54)	0.91 (0.46–1.83)
≥5 years	881	0.86 (0.66–1.13)	1.08 (0.89–1.31)	1.06 (0.85–1.32)	1.22 (0.92–1.63)	1.06 (0.87–1.29)	0.56 (0.32–0.97)
*P*-value for trend^b^		0.057	0.079	<0.0001	0.354	0.672	0.328

HDL cholesterol: high-density lipoprotein cholesterol.

Multivariate-adjusted relative odds ratios (95% confidence interval) are shown.

Cigarette smoking status and daily cigarette consumption were adjusted for age, eating habits score, regular exercise, and daily alcohol consumption.

Years since cessation of smoking was adjusted for age, eating habits score, regular exercise, cigarettes smoked per day (in ex-smokers), and alcohol consumption status.

^a^The test for trend was calculated across increasing categories of daily cigarette consumption for current smokers only.

^b^The test for trend was calculated across increasing categories of years after cessation for ex-smokers only.

Table 4 shows that current alcohol consumption was significantly associated with the prevalence of MetS (*P* = 0.030 for trend). The multivariate-adjusted odds ratio for the prevalence of MetS in those who consumed ≥69 grams ethanol per day was 1.54 (95% CI, 1.06 to 2.23), with nondrinkers as reference. Current alcohol consumption was significantly associated with the prevalences of high waist circumference, high triglycerides and hypertension. In addition, the odds ratio of the prevalence of low HDL cholesterol decreased as current alcohol consumption increased.

**Table 4.** Adjusted odds ratios of the components of metabolic syndrome according to alcohol consumption status

	*n*	Metabolic syndrome	High waist circumference	High triglycerides	Low HDL cholesterol	Hypertension	High fasting plasma glucose
Alcohol consumption status							
Nondrinker	795	1.00	1.00	1.00	1.00	1.00	1.00
Ex-drinker	249	1.25 (0.84–1.87)	1.28 (0.95–1.73)	1.15 (0.83–1.59)	1.65 (1.15–2.36)	1.00 (0.72–1.39)	1.34 (0.66–2.72)
Current drinker	2860	1.08 (0.85–1.38)	1.10 (0.93–1.31)	1.06 (0.88–1.28)	0.70 (0.56–0.89)	1.58 (1.31–1.90)	1.07 (0.69–1.67)

Daily alcohol consumption							
Nondrinker	795	1.00	1.00	1.00	1.00	1.00	1.00
0.1–22.9 grams/day	1246	1.01 (0.77–1.33)	0.99 (0.82–1.20)	0.96 (0.77–1.18)	0.90 (0.70–1.18)	1.14 (0.93–1.41)	0.94 (0.61–1.57)
23–45.9	885	1.05 (0.79–1.40)	1.13 (0.92–1.39)	0.97 (0.78–1.22)	0.57 (0.42–0.77)	1.79 (1.43–2.23)	1.15 (0.68–1.92)
46–69	450	1.11 (0.79–1.54)	1.21 (0.94–1.54)	1.18 (0.91–1.54)	0.59 (0.41–0.85)	2.09 (1.61–2.72)	1.02 (0.55–1.91)
≥69	279	1.54 (1.06–2.23)	1.54 (1.15–2.05)	1.80 (1.33–2.43)	0.47 (0.29–0.74)	3.09 (2.27–4.19)	1.51 (0.77–2.96)
*P*-value for trend^a^		0.030	0.001	<0.0001	0.001	<0.0001	0.190

Years since cessation of alcohol consumption							
Nondrinker	795	1.00	1.00	1.00	1.00	1.00	1.00
<1 year	17	1.33 (0.29–6.21)	1.12 (0.38–3.27)	0.67 (0.18–2.51)	3.05 (0.99–9.34)	0.89 (0.24–3.36)	0.99 (0.70–1.24)
1–4 years	91	0.99 (0.49–1.98)	1.22 (0.76–1.97)	0.74 (0.41–1.34)	1.30 (0.71–2.38)	0.86 (0.50–1.49)	0.79 (0.18–3.52)
≥5 years	141	1.39 (0.85–2.29)	1.41 (0.94–2.12)	1.06 (0.68–1.65)	1.50 (0.94–2.39)	1.19 (0.78–1.82)	0.75 (0.85–1.41)
*P*-value for trend^b^		0.246	0.227	0.161	0.717	0.269	0.756

HDL cholesterol: high-density lipoprotein cholesterol.

Multivariate-adjusted relative odds ratios (95% confidence interval) are shown.

Alcohol consumption status and daily alcohol consumption were adjusted for age, eating habits score, regular exercise, and cigarettes smoked per day.

Years since cessation of alcohol consumption was adjusted for age, eating habits score, regular exercise, daily alcohol consumption (in ex-drinkers only), and cigarette smoking status.

^a^The test for trend was calculated across increasing categories of daily alcohol consumption for current drinkers only.

^b^The test for trend was calculated across increasing categories of years since cessation in ex-drinkers only.

The relationship of the prevalence of MetS with cigarette smoking or alcohol consumption cessation was not statistically significant (Tables 3, 4); however, the greater the number of years since cessation, the lower the odds ratio of high triglycerides (*P* < 0.0001 for trend).

Table 5 shows the prevalence of MetS according to the combination of cigarette smoking and alcohol consumption. With participants who had neither smoked nor drank alcohol as reference, the odds ratios of the prevalence of MetS in subjects who smoked ≥30 cigarettes per day for each category of alcohol consumption (nondrinker, <69 grams ethanol per day, ≥69 grams ethanol per day) were 2.14 (95% CI, 1.05 to 4.36), 1.97 (1.23 to 3.16), and 3.63 (1.91 to 6.90), respectively, indicating a significant association with the prevalence of MetS. The interaction of smoking ≥30 cigarettes per day with drinking ≥69 grams ethanol per day was 2.03 (1.02 to 4.01, *P* = 0.043).

**Table 5.** Adjusted odds ratios of metabolic syndrome according to combined cigarette smoking and alcohol consumption status

		Nondrinker	Alcohol consumption	

			0.1–68.9 grams/day	≥69
Nonsmoker	*n*	397	788	26
	MetS	43 (10.8%)	90 (11.4%)	4 (15.4%)
	OR	1.00 reference	1.00 (0.67–1.48)	1.10 (0.36–3.37)
Daily cigarette consumption				
1–29 cigarettes/day	*n*	186	699	80
	MetS	27 (14.5%)	89 (12.7%)	12 (15.0%)
	OR	1.22 (0.72–2.08)	1.00 (0.67–1.49)	1.12 (0.55–2.26)

≥30	*n*	56	190	59
	MetS	13 (23.2%)	45 (23.7%)	20 (33.9%)
	OR	2.14 (1.05–4.36)	1.97 (1.23–3.16)	3.63^a^ (1.91–6.90)

The number of subjects with metabolic syndrome and multivariate-adjusted relative odds ratios (95% confidence interval) are shown.

Adjusted for age, eating habits score, and regular exercise.

The test excluded ex-smokers and ex-drinkers.

^a^Interaction = 2.03 (1.02–4.01), *P* = 0.043; the reference was smoking 1 to 29 cigarettes per day and consumption of 0.1 to 68.9 grams of ethanol per day.

## DISCUSSION

In this cross-sectional study, we observed that cigarette smoking and alcohol consumption were significantly associated with the prevalence of MetS in Japanese men, after adjusting for age and other potential confounding factors. In particular, the combination of heavy smoking and heavy drinking was strongly associated with the prevalence of MetS.

### Relationship between cigarette smoking and MetS

Previous studies in Japanese men reported a relationship between cigarette smoking and MetS.^5^^–^^7^ Nakanishi et al found a positive dose-response relationship between the daily number of cigarettes smoked and the risk of MetS in both cross-sectional and longitudinal analysis.^7^ We, too, observed that cigarette smoking was closely linked to MetS.

In the present study, current smoking was significantly associated with a high waist circumference—a necessary component for a diagnosis of MetS—as well as with high triglycerides and low HDL cholesterol. In logistic regression analysis, the multivariate-adjusted odds ratio of a hemoglobin A1c ≥5.2% in current smokers was 1.30 (95% CI, 1.08 to 1.56; data not shown). A possible mechanism for the increased risk of MetS due to cigarette smoking is that smoking causes higher fasting plasma cortisol concentrations, resulting in an increase in visceral adipose tissue.^26^ Smoking also causes deterioration of lipid metabolism via decreased lipoprotein lipase.^27^ In addition, some studies have demonstrated that smokers are insulin-resistant and hyperinsulinemic.^27^^,^^28^ Smoking is reported to cause systemic oxidative stress. Increased oxidative stress in accumulated fat is the underlying cause of the dysregulation of adipocytokines, which leads to insulin resistance.^29^ Our findings support the hypothesis that cigarette smoking is independently related to the prevalence of MetS components.

We observed that the odds ratio of the prevalence of hypertension was lower in current smokers than in nonsmokers. We could not identify any confounding by age, obesity, or alcohol consumption on the relation between cigarette smoking and blood pressure. Some studies reported lower blood pressure among smokers than nonsmokers.^30^^,^^31^ This may be due to the depressing effect of smoking on the myocardium, which potentially results in reduced cardiac output and, consequently, lower blood pressure.^30^ One study found that blood pressure was inversely related to serum cotinine concentration in smokers^31^; however, the blood pressure of smokers at general health check-ups was lower than usual because it was measured during a brief cessation of smoking.^32^^,^^33^ Recent cross-sectional studies showed that smoking was not associated with blood pressure.^34^^,^^35^ In this study, we did not address the difference in blood pressure measured in usual settings and at annual health check-ups. Neither did we record the time elapsed since the most recent smoking episode. This may explain why we found no association of smoking with blood pressure.

We found no association between the prevalence of MetS and the length of time since smoking cessation. In ex-smokers who had previously smoked ≥30 cigarettes per day, the multivariate-adjusted odds ratio for the prevalence of MetS was 1.64 (1.14 to 2.38) for those who had quit less than 10 years before, and 0.74 (0.45 to 1.19) for those who had quit more than 10 years before (data not shown). Thus, the harmful association of heavy smoking with MetS appears to last longer than expected after smoking cessation.

### Relationship between alcohol consumption and MetS

Alcohol consumption was significantly associated with the prevalence of MetS, and with 4 of the 5 components of MetS, ie, waist circumference, triglycerides, HDL cholesterol, and hypertension. Some studies have reported that the association of alcohol consumption with MetS may be explained by relations with the components of MetS.^10^^,^^36^ An association of elevated HDL cholesterol concentration with moderate alcohol intake has been reported,^37^^,^^38^ and this association is believed to be a key factor in the widely accepted cardioprotective effect of alcohol.^12^ In addition, the associations of light-to-moderate alcohol consumption with plasma indicators of thrombopoiesis are well established.^39^ In regard to the influence of drinking on health, it is clear that alcohol consumption has both positive and negative effects.^18^

The association of the prevalence of MetS with the length of time after cessation of alcohol consumption was not statistically significant in this study. We suspected that one reason for cessation of alcohol consumption was illness, so we excluded 5 subjects from the analysis: 1 with hepatitis B, 2 with hepatitis C, and 2 with abnormal liver function. The results nevertheless showed a similar trend after exclusion of these participants (data not shown). Moreover, our study revealed no significant dose relationship between alcohol consumption (<69, ≥69 grams/day) in ex-drinkers and the present prevalence of MetS. This was most likely due to the fact that most ex-drinkers consumed <69 grams ethanol per day. Thus, we were unable to obtain significant findings about the association between years since cessation of alcohol consumption and MetS.

### Relationship between MetS and the combination of cigarette smoking and alcohol consumption

Our study demonstrated that the combination of cigarette smoking and alcohol consumption was significantly associated with the prevalence of MetS. Kanbe et al showed that combined current smoking and drinking was associated with a higher triglyceride level^19^; however, 1 study reported no interactive effect of smoking and drinking on any laboratory test.^18^ With regard to daily cigarette consumption and alcohol consumption, it is noteworthy that the combination of heavy smoking and heavy drinking was shown to be a potential risk factor of MetS. As compared with nonsmokers, male smokers exercised less regularly, drank more alcohol, consumed more salty foods, and ate more quickly.^14^^,^^15^ Furthermore, male daily drinkers consumed a greater number of salty foods, more fat, and less fruit.^10^^,^^14^ In the present study, participants who were heavy smokers and heavy drinkers skipped breakfast more often, had higher scores for salt and fat consumption, and were more obese (data not shown). The reason for the marked increase in the odds ratio for this combination, which was particularly prominent in heavy smokers, appears to be the synergism of accumulated unhealthy behaviors.

The strength of our study was that we showed an independent relationship of cigarette smoking and alcohol consumption with MetS. Furthermore, we found that concomitant heavy smoking and drinking were associated with a higher prevalence of MetS.

The study does, however, have several limitations. First, cross-sectional observations cannot provide any evidence of causal associations. Second, we must admit the possibility of recall bias, particularly concerning the number of cigarettes smoked in ex-smokers and alcohol consumption in ex-drinkers. Longitudinal research with a larger number of subjects is needed to delineate more precisely the effect of cigarette smoking and alcohol consumption on the prevalence of MetS.

In conclusion, this cross-sectional analysis indicates that cigarette smoking and alcohol consumption are independently related to the prevalence of MetS, and that the prevalence of MetS was higher among Japanese men who smoked and drank heavily. However, these causal relationship need to be confirmed in longitudinal studies.
